# Prediction of prostate cancer Gleason score upgrading from biopsy to radical prostatectomy using pre-biopsy multiparametric MRI PIRADS scoring system

**DOI:** 10.1038/s41598-020-64693-y

**Published:** 2020-05-07

**Authors:** Saeed Alqahtani, Cheng Wei, Yilong Zhang, Magdalena Szewczyk-Bieda, Jennifer Wilson, Zhihong Huang, Ghulam Nabi

**Affiliations:** 1 Division of Imaging Sciences and Technology, School of Medicine, Ninewells Hospital, University of Dundee, Dundee, UK; 2 School of Science and Engineering, University of Dundee, Dundee, UK; 3 Department of Clinical Radiology, Ninewells Hospital, Dundee, UK; 4 Department of Pathology, Ninewells Hospital, Dundee, UK; 5 Department of Radiological sciences, college of applied medical science, Najran University, Najran, Saudi Arabia

**Keywords:** Prostate cancer, Cancer imaging, Prostate

## Abstract

An increase or ‘upgrade’ in Gleason Score (GS) in prostate cancer following Transrectal Ultrasound (TRUS) guided biopsies remains a significant challenge to overcome. to evaluate whether MRI has the potential to narrow the discrepancy of histopathological grades between biopsy and radical prostatectomy, three hundred and thirty men treated consecutively by laparoscopic radical prostatectomy (LRP) between July 2014 and January 2019 with localized prostate cancer were included in this study. Independent radiologists and pathologists assessed the MRI and histopathology of the biopsies and prostatectomy specimens respectively. A multivariate model was constructed using logistic regression analysis to assess the ability of MRI to predict upgrading in biopsy GS in a nomogram. A decision-analysis curve was constructed assessing impact of nomogram using different thresholds for probabilities of upgrading. PIRADS scores were obtained from MRI scans in all the included cases. In a multivariate analysis, the PIRADS v2.0 score significantly improved prediction ability of MRI scans for upgrading of biopsy GS (p = 0.001, 95% CI [0.06–0.034]), which improved the C-index of predictive nomogram significantly (0.90 vs. 0.64, p < 0.05). PIRADS v2.0 score was an independent predictor of postoperative GS upgrading and this should be taken into consideration while offering treatment options to men with localized prostate cancer.

## Introduction

Histology from biopsies categorised into Gleason score is the only confirmatory test for cancer diagnosis and is most commonly used for risk stratification of men with a recent diagnosis of prostate cancer. Based on this men are counselled for various treatment options. MR imaging data is not considered in risk stratification at present. With increasing therapeutic options available to men with a diagnosis of prostate cancer, scrutiny of information from biopsy grade becomes increasingly important. There is around 35.5% (range: 14–51%) upgrading of biopsy GS on LRP^1^. Many factors contribute to the discrepancy between needle biopsy and corresponding radical surgery GS. Under calling of Gleason cribriform Gleason pattern 4 as pattern 3 or the presence of borderline grades due to barely appreciable glandular differentiation under microscope and lack of sampling of tertiary grade disease on biopsies are known contributors. Factors such as age, size of prostate, extent of cancer on biopsy needle and number of biopsy samples (extended/ or mapping) have also been known to impact on the incidence of upgrading^2^.

In light of a number of studies reporting upgrading or under-grading of prostate cancer on needle biopsies, there is the potential for under treatment or overtreatment (i.e. radiotherapy and hormone duration). Several publications^3,4^ and consensus updates on the Gleason grading system have partially addressed this issue including recommendation of deriving GS by adding the most common and highest Gleason pattern on biopsy rather than original method of adding the primary and second most common patterns^5^. Moreover, upgrading if suspected, has long-term outcome implications. Corcoran *et al*.^6^ have shown that even after adjusting for known preoperative variables (including clinical stage, prostate-specific antigen (PSA), number of positive cores and percentage of positive cores) upgrade to a higher Gleason Score (GS) remained a strong and independent predictor of biochemical recurrence after attempted local curative therapy, this underscores the importance of gaining more information to predict upgrading of biopsy GS in men diagnosed with prostate cancer as this may serve as a marker of biologically aggressive disease.

Pre-biopsy MRI has recently been shown to hold great promise in the detection and characterisation of prostate cancer^7^. A negative scan (no lesion seen on the MRI scan) showed a high negative predictive value for the presence of significant prostate cancer^8^. Song *et al*.^9^ reported a high predictive value of PIRADS v2 in predicting upgrading of GS from biopsy, however this study was retrospective and MR Imaging was obtained at least 3 weeks following biopsies - an approach known to impact interpretation of images. Post-biopsy haemorrhage is the most common false-positive finding for prostate cancer^10^. In this study, there was no attempt to align histopathological sectioning to MRI using recently reported 3D-mould technology. Therefore, this is the first report describing predictive accuracy of pre-biopsy MRI in upgrading biopsy GS following LRP using patient-specific 3D moulds to ensure permitted alignment of excised prostates with MRI scans.

## Patients and methods

### Study population

**Figure 1:**
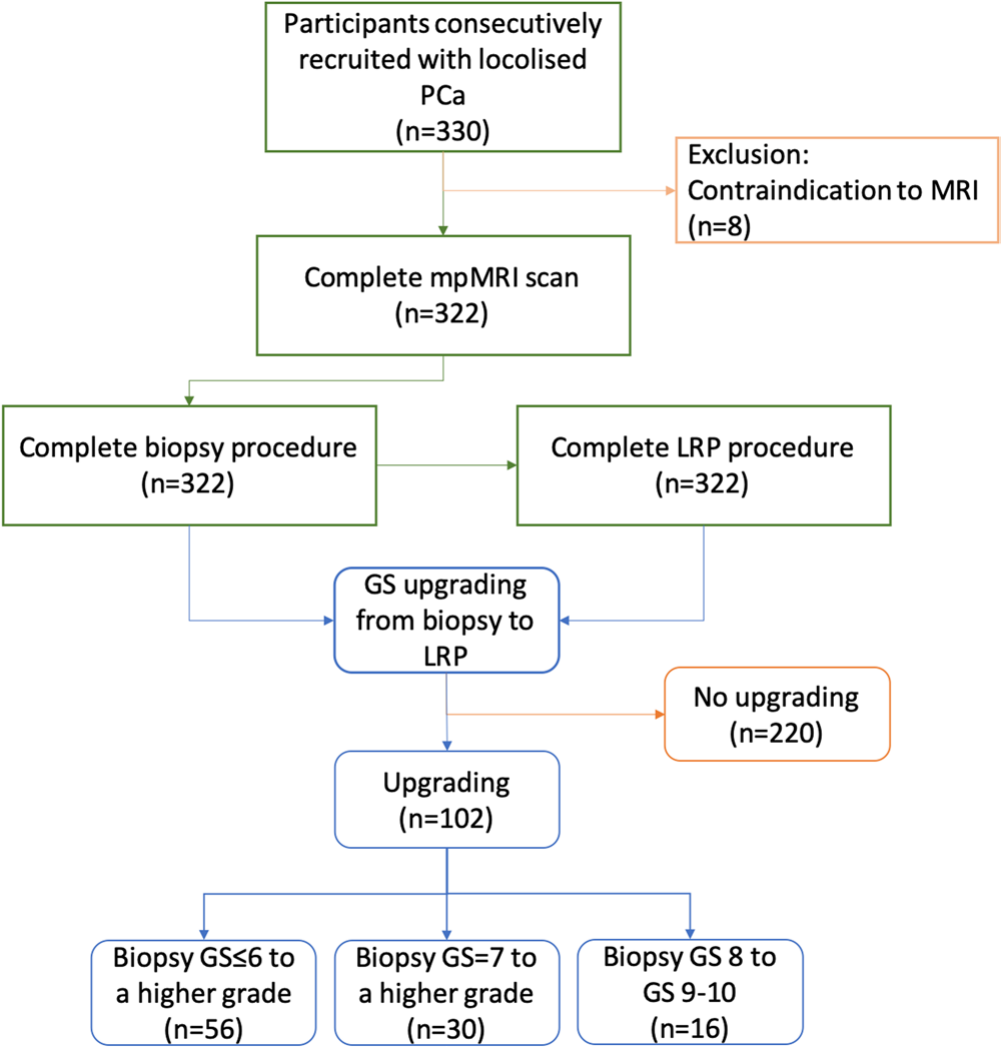
Flowchart of the study.

This is a study with prior Caldicott institutional approval (Caldicott/IGTCAL5626). All experiments including the study protocol study followed approved institutional guidelines. The study had ethical approval (14/ES/1070) with each participant informed consenting to the use of their imaging data. Between July 2014 and January 2019, 330 men consecutively treated by LRP who were diagnosed with localised prostate cancer with raised PSA or/and abnormal digital rectal examination were included in this study. They were offered mpMRI and those with positive MRI results (PIRADS score 3 and above) were performed transrectal ultrasound (TRUS)-guided prostate biopsy (12 cores) followed. Of these patients, eight were excluded because of contraindication to MRI such as a heart pacemaker and metallic foreign body including three claustrophobic patients. Further analysis included remaining 322 patients (Fig. 1). The clinical, pathological and imaging factors information of the patients, including age, weight, preoperative PSA, PSA density, number of positive cores, maximum percentage of cancer per core and PIRADS v2 score on multiparametric MRI (mpMRI) were recorded. GS upgrading defined as a biopsy GS increasing from lower to higher grade on reported before^2^. Table 1 summarises the baseline characteristics between upgraded and non-upgraded groups of the cohort.

**Table 1:** Patient characteristics.

Number of patients	Total	Upgrading	No upgrading
	322	102	220
Age (y), mean ± SD (range)	66.83 ± 5.9(44–77)	66.82 ± 6.12(49–77)	66.85 ± 5.9(44–77)
Prostate Weight mean ± SD (range)	63.7 ± 30.13(12–207)	65.3 ± 26.2(20–155)	63.1 ± 31.78(12–207)
PSA level (ng/ml), mean ± SD (range)	11.1 ± 7.39(0.1–47.7)	12.6 ± 9.98 (2–47.7)	10.39 ± 5.7(0.1–41)
PSA Density (ng/ml^2^), mean ± SD (range)	0.261 ± 0.234 (0.001–3.48)	0.212 ± 0.183(0.035–1.11)	0.203 ± 0.254(0.00198–3.48)
Number of positive cores	4.8 ± 3.4(1–12)	4.1 ± 3.07(1–12)	5.08 ± 3.42(1–12)
Maximum percentage of cancer per core	50.2 ± 30.4(5–100)	42.5 ± 30.6(5–100)	53.3 ± 29.52(5–100)
PIRADS from mpMRI Benign (1,2)	17 (5%)	4 (4%)	13 (6%)
PIRADS 3	21 (7%)	6 (6%)	15(7%)
PIRADS 4	78 (24%)	26 (26%)	52 (23%)
PIRADS 5	206 (64%)	66 (65%)	140 (64%)

### Hypotheses of the study

We hypothesised that pre-biopsy MRI with PIRADS classification of suspicious area in prostatic cancer improve prediction of GS upgrading from biopsy to radical prostatectomy. Upgrading of GS on histology was defined as change of GS from lower to higher grade between biopsy and histology from radical prostatectomy.

### MRI protocol and PIRADS score

All patients’ mpMRI scans were performed on 3 T scanner (TIM Trio, Siemens, Erlangen, Germany) 2 weeks before TRUS-guided biopsies. The mpMRI protocol was derived from the European Society of Uro-radiology Guidelines 2012 for the detection of prostate cancer and the subsequent publication of version 2^11^. Table 2 briefly summarizes the MRI acquisition parameters. Localiser images were acquired in all three imaging planes, whereby the plane of the prostate was defined in relation to the rectal wall.

**Table 2:** MRI acquisition parameters.

	T1WI		High resolution T2WI			DWI	DCE
	Axial	Sagittal	Axial	Coronal	DWI	DWI high b-value	Dyn Gd-MRI
TR (ms)	650	6000	4000	5000	3300	3300	4.76
Sequence	2DTSE	2DTSE	2DTSE	2DTSE	2DEPI	2DEPI	3D VIBE
TE (ms)	11	102	100	100	95	95	2.45
Flip angle (°)	150	140	150	150	—	—	10
Slice thickness (mm)	3	3	3	3	3	3	3
Slice gap (mm)	0.6	0.6	0.6	0.6	0	0	0.6
Resolution (pixels)	320	320	320	320	192	192	192
FOV (mm)	200	200	200	200	280	280	280
b-values (s/mm^2^)	—	—	—	—	50,100,500,1000	2000	—
Temporal resolution (s)	—	—	—	—	—	—	4

**Figure 2:**
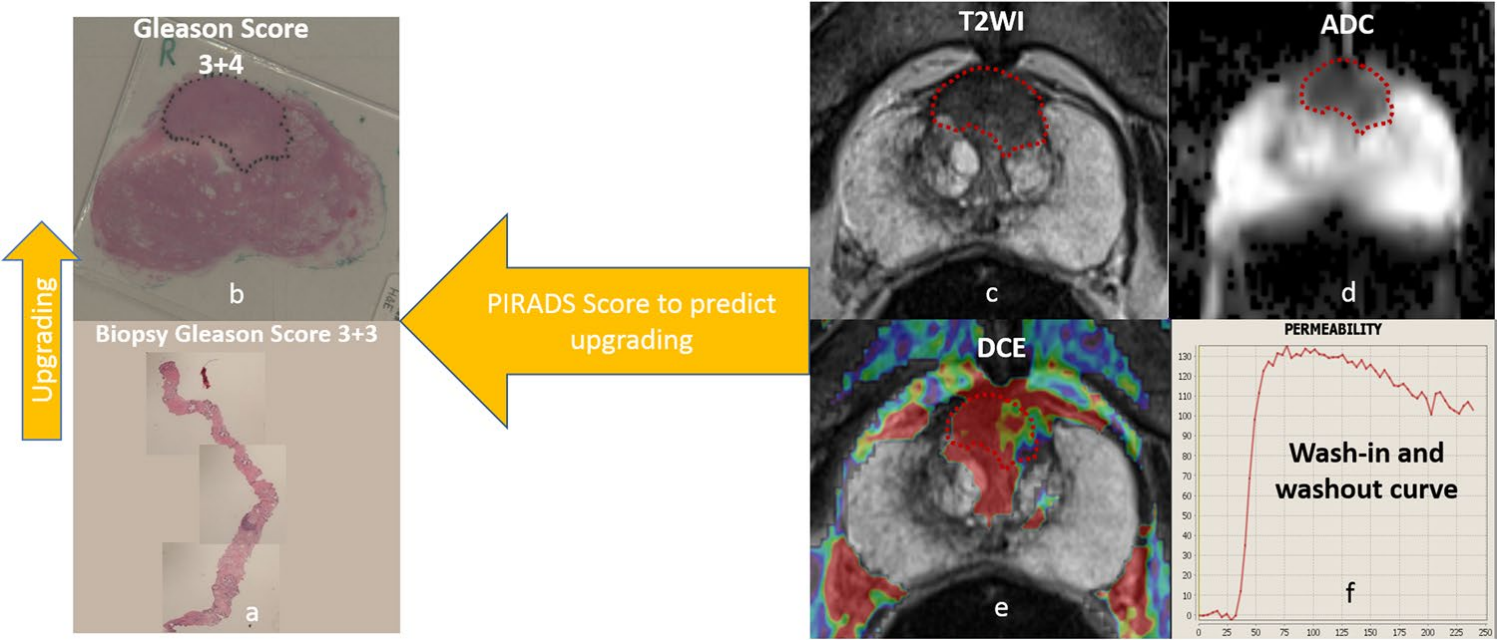
(**a**) A 73 year-old man with Gleason score 6 disease on prostate cancer on TRUS-Guided biopsies. (**b**) The grade was upgrading to GS 7 on whole mount radical prostatectomy specimen (**c**) Axial T2-wighted image shows ill-defined homogeneous low-signal-intensity on the central zone (**d**), Apparent diffusion coefficient (ADC) shows restricted diffusion in low-signal mass and (**e**) dynamic contrast enhanced (DCE) shows fast and strong enhancement and early contrast agent washout (type 3 curve), (**e,f**) The lesion was scored as PIRADSv2 5 (>1.5 cm) and based on parameters described here.

The mpMRI images were analysed and scored by experienced uro-radiologists using PIRADS v2.0; and the radiologists were blinded to all patients’ pathology results. PIRADS v2.0 assessment categories were described as follows: score 1, clinically significant cancer is highly unlikely to be present; score 2, clinically significant cancer is unlikely to be present; score 3, the presence of clinically significant cancer is equivocal; score 4, clinically significant cancer is likely to be present; and score 5, clinically significant cancer is highly likely to be present **(**Fig. 2**)**.

### Histopathology data and analysis

The biopsy results were analysed by experienced pathologists; who were blinded to MRI findings. The GS for each patient was obtained. The radical prostate specimens for histology were sliced in patient-specific moulds to aid orientations between imaging and histology, which were fabricated using a 3D printer as described by our group and others previously^12,13^. Specifically, patient specific 3D printed moulds were made prior to surgery based on the T2-weighted MRI prostate capsule the moulds were customised for each patient using MIMICS and Solidworks. Moulds were printed at 200 micro resolution using a consumer grade 3D printer (MakerBot Replicator 5^th^ generation). The average mould required 120 minutes to design, 4 to 7 hours to print and an expense for materials of less than $7^12^.

### Statistical analysis

Baseline characteristics of patients and pathological outcomes were compared using a chi-square test for categorical data (PIRADS score) and a Student t-test or ANOVA for continuous data (age, weight, PSA level, PSAD, number of positive cores and maximum percentage of cancer per core). Univariate logistic regression was applied to investigate the association of clinical variables with the upgrading of biopsy GS. Variables with P < 0.05 in the univariate analysis were further assessed using a multivariate logistic regression analysis to identify factors predictive of GS upgrading. In order to estimate the area under the curve (AUC) for predicting GS upgrading to determine the diagnostic performance of clinical variables with or without PIRADS score, a receiver operating characteristic (ROC) curve analysis was conducted.

In addition, logistic regression model coefficients were used to perform a nomogram predicating the probability of GS upgrading. Non-informative or non-significant variables in univariate logistic regression for GS upgrading were removed. The value of concordance indexes (c-index) were calculated and compared. The bias-corrected calibrated values were generated from internal validation based on 200 bootstrap resamples.

A decision-analysis curve was constructed assessing impact of nomogram using different thresholds probabilities of upgrading (none of the GS upgrade to all GS upgrade).

Analyses were performed using SPSS 22 (IBM Corporation, New York, US) and R software. The alpha level was set at 0.05 to determine two-tailed significance.

## Results

### Upgrading cohort characteristics

In total, 322 men were included in our study. Table 3 shows the concordance between the biopsy and prostatectomy Gleason score sums. Of these, (102/322; 31.6%) had GS upgrading from biopsy to LRP. Almost half of this upgrading was from biopsy GS ≤ 6 disease (56/102; 55%). More than half of whole cohort (175/322; 54%) had a GS 7 on prostate biopsy and (30/175; 17%) men had GS upgrading. Finally, eighty five of the cohort (85/322; 26%) had a GS ≥ 8 on prostate biopsy and (16/85; 18.8%) men had GS upgrading from GS 8 on prostate biopsy to GS > 8 at LRP.

**Table 3:** Comparison between biopsy and radical prostatectomy Gleason score sum.

Biopsy Gleason sum	Radical prostatectomy Gleason sum				Total
	6	7	8	9–10	
1–5	0	3	0	1	4
6	6	46	2	4	58
7	0	145	14	16	175
8	0	19	9	16	44
9–10	0	2	3	36	41
Total	6	215	28	73	322

**Figure 3:**
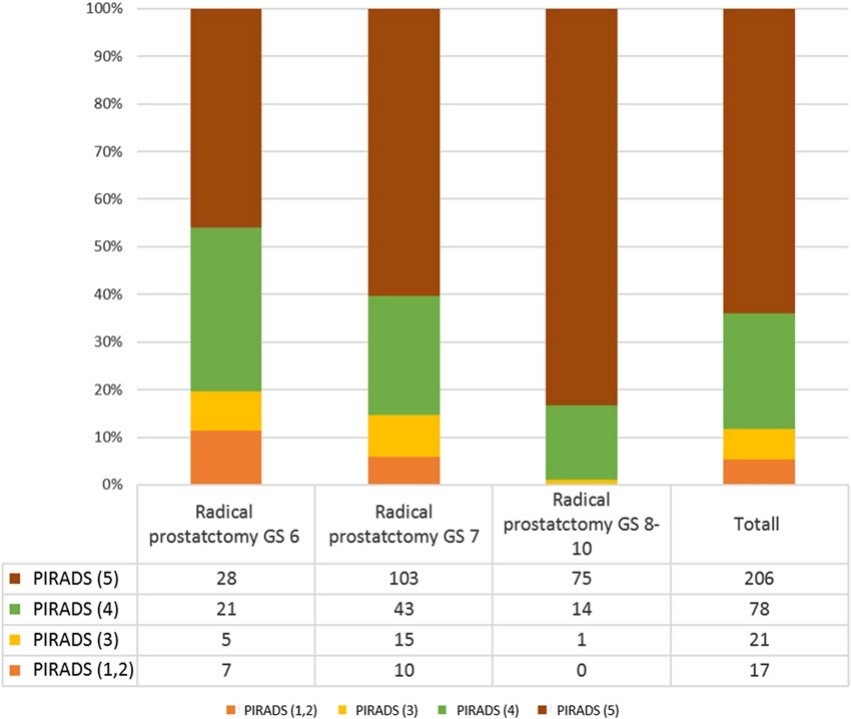
Radical prostatectomy Gleason score stratified according to PIRADS score.

The correlation between PIRADS score and pathologic GS at LRP is demonstrated in Fig. 3. Of the 322 patients, the distribution of PIRADS score was as follows: score 1 and 2 in 17 (17/322; 5%) patients, score 3 in 21 (21/322; 7%) patients, score 4 in 78 (78/322; 24%) patients, and score 5 in 206 (206/322; 64%) patients.

### Predictions of GS upgrading

Table 4 shows the outcomes of the logistic regression analysis and predictive variables of GS upgrading. On univariate analyses, increased preoperative PSA levels, number of positive cores, maximum percentage of cancer per core and PIRADS ≥ 4 were all significantly associated with GS upgrading (p < 0.05). Age, weight of prostate and PSAD did not show any significance (p > 0.05) which were excluded from further analyses. In the multivariate analyses, PIRADS ≥ 4 and higher PSA level were both statistically significant and independently predictive of GS upgrading (p = 0.001 and 0.003, respectively).

**Table 4:** Univariate and multivariate logistic regression analysis.

	Univariate		Multivariate	
	OR (95% CI)	*P*-value	OR (95% CI)	*P*-value
Age	1.005 (0.962–1.041)	0.799		
Pathology weight	1.002(0.931–1.009)	0.540		
Number of positive cores	0.86(0.87–0.96)	0.005	0.970(0.98–1.01)	0.69
PSA level (ng/ml)	1.040 (1.009–1.073)	0.001	1.09(1.030–1.160)	0.003
PSA Density (ng/ml^2^)	1.15 (0.44–3.04)	0.76		
Maximum percentage of cancer per core	0.988 (0.980–0.96)	0.002	0.970 (0.84–1.12)	0.62
PIRADS				
≤ 3	1 (reference)	—	1 (reference)	—
> 3	0.017(0.08–0.04)	0.001	0.014 (0.06–0.034)	0.001

**Figure 4:**
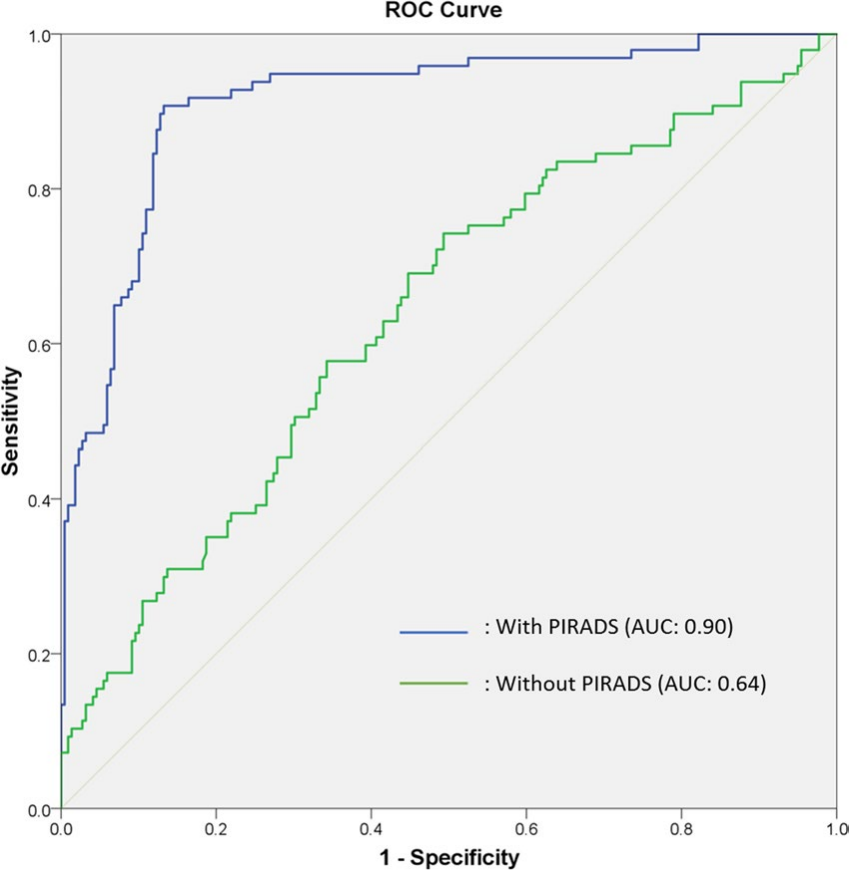
Receiver operating characteristic (ROC) curve of the clinical variables with and without PIRADS score.

In Fig. 4, PIRADS v2 score with PSA value show a higher accuracy than PSA alone for predicting GS upgrading (AUC = 0.90 and 0.64, respectively, p < 0.001).

### Impact of PIRADS score on prediction of GS upgrading in relation to other factors

**Figure 5:**
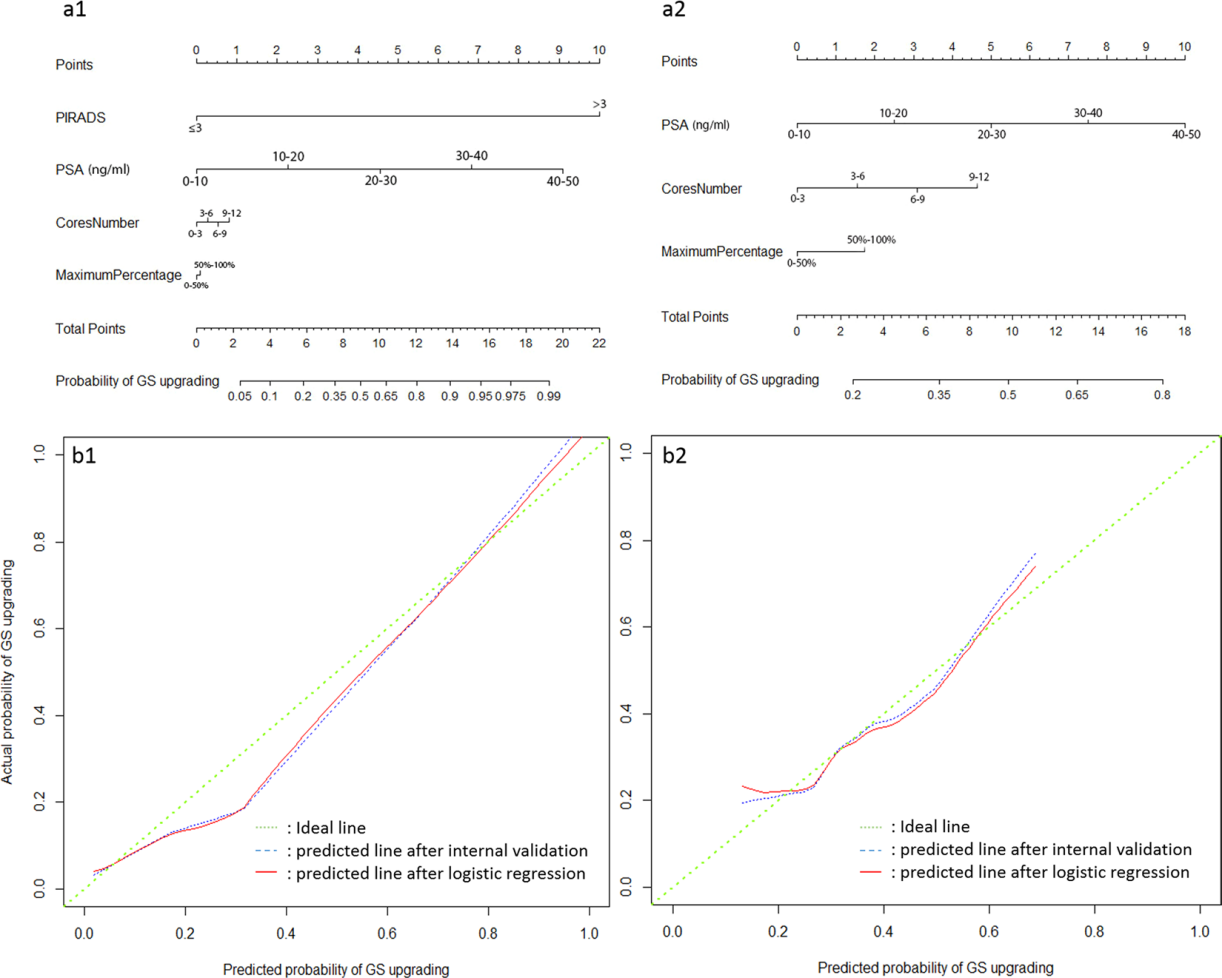
The nomograms of Gleason score upgrading prediction with (a1) and without PIRADS score (a2). Calibration plots of observed and predicted probability of GS upgrading with (b1) and without PIRADS score (b2).

Figure 5a_1_,a_2_ show the nomograms constructed for upgradation of biopsy GS with and without PIRADS v2 score data. Longer scales indicated higher percentage of impact and larger points were suggesting probability of upgrading. PIRADS score had the greatest impact followed by PSA level.C-index of the established nomogram which had PIRADS v2 score variable to predict the GS upgrading in the cohort was significantly higher than that of the nomogram without PIRADS score (0.90 [95% CI 0.87–0.89] vs. 0.64 [95% CI, 0.57–0.70], p = 0.001). The nomograms were then validated using 200 bootstrap samples, internal calibration curves are shown in Fig. 5b_1_,b_2_.

### Decision curve analysis

**Figure 6:**
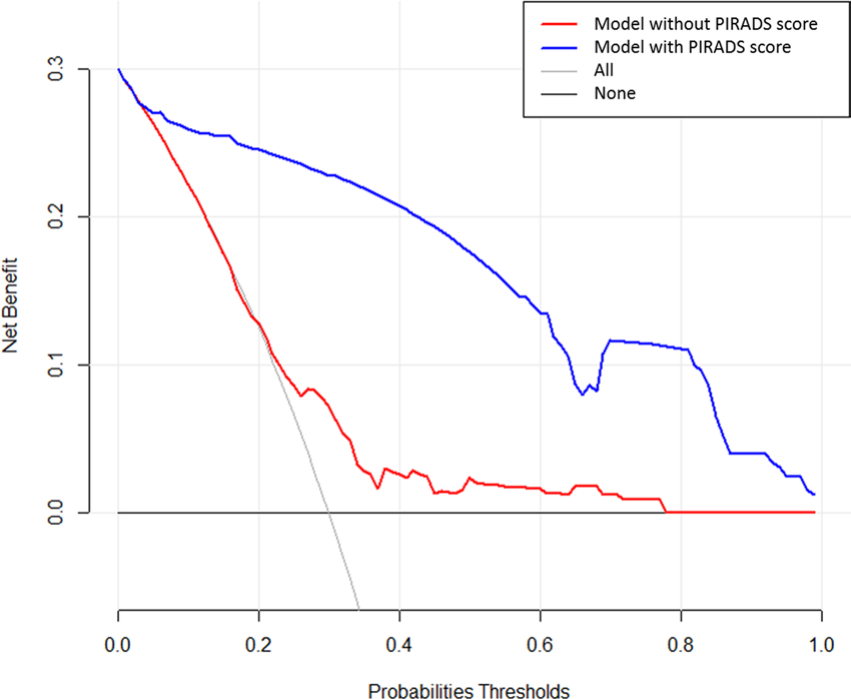
Decision analysis demonstrated a high net benefit of PIRADS score model across a wide range of threshold probabilities. Prediction model without PIRADS score (red line); prediction model with PIRADS score (blue line).

The decision analysis curve is shown in Fig. 6. The net benefit for the model using PIRADS score was significantly higher at all thresholds compared with the model without PIRADS score. As seen in Fig. 6, the decision curve line (depicted by a red line) of the model without the PIRADS scores remained close to the line with threshold probabilities ranging from 0 to 0.25. In contrast, a higher positive net benefit was obtained in the range of threshold probabilities ranging from 0.05 to 1.0 in the model with PIRADS scores.

## Discussion

### Principal findings of the study

This is the first study to bring together information of PIRADS scores in pre-biopsy MRI and an improved image oriented histopathological grossing of RP specimen by making the use of the mould, bridging the gap in the existing literature. Our results indicate a significant advantage (C-index 0.90 vs 0.64) of using the prediction model including PIRADS scores added to conventional clinic-pathological characteristics (PSA level, percentage of cancer on core-biopsies, gland size etc.) of men with prostate cancer confirmed by systematic transrectal random biopsies relative to a model without PIRADS scores. Prostate biopsy GS upgrading remains a challenge for physicians managing localised prostate cancer, as better knowledge of contributing factors and how to narrow the gap is lacking^2^. To inform any consensus, we need an improved understanding of the role imaging can play, in particular pre-biopsy MRI, in predicting GS change and adverse downstream oncological outcomes. Although, recent improvements have been made in refining biopsy strategies and in reducing sampling errors, a significant and continued effort is still needed to identify men at risk of GS upgrading.

### Study findings in context of the reported literature

Wang JY *et al*.^14^ reported a nomogram with C-index of 0.795 using preoperative factors without imaging data in a non-screened population from China. This is similar to our study as the healthcare system for the cohort reported here did not have men screened for prostate cancer. Table 5 shows the predictive ability of various reported nomograms in upgrading of biopsy GS of prostate cancer in screened populations^1,14–17^. The upgrading rate of biopsy GS seen in our cohort is similar to a larger cohort of 2982 patients reported previously^15^. A higher percentage of men with a GS of 6 were upgraded in the present study. It is interesting that despite the higher number of cores obtained in the present study (12 in number) in comparison with 6–10 biopsy cores obtained in study by Chun FK *et al*.^15^; upgrading rates remain comparable.

**Table 5:** literature review and comparison between previous and current studies.

Author	year	Number of patients	Performance (C-index)	Significant parameters on multivariate analysis
Chun, FK	2006	2982	0.804	PSA level, clinical stage and primary and secondary GS
Kulkarni, GS	2007	175	0.71	PSA level and the level of pathologist expertise
Budäus, L	2010	414	0.708	PSA level, clinical stage, prostate volume and percent of positive cores
Wang, JY	2014	220	0.789	PSA level, clinical stage, and primary and secondary GS
Biming, He	2016	411	0.753	Primary and secondary GS and obesity
This study	2019	322	0.90	PSA level and PIRADS score on mp-MRI

A number of previous studies have carried out multivariate analyses of factors responsible for upgrading of biopsy GS including construction of nomograms (Table 5). In predictive oncology, nomograms have huge potential to help clinicians determine the risk of disease progression and identify those who would experience a greater benefit from multimodality therapy. This approach may result in avoiding unnecessary treatment and improve quality of life by reducing side effects of therapy through better and more precise approach. However, a careful approach is needed to construct nomogram based on specific question, the study population, the method of construction, and its ability to apply to a particular clinical situation. We have followed guidelines described in previous publications^18–20^ in constructing nomogram in this study including selection of variables and statistical methods. The nomogram in the present study has been internally validated (Cross-validation and bootstrapping). External validation of nomogram was not carried out in the present study as this would require further prospective multi-centre recruitment of a cohort. Since D’Amico pioneered this approach^21^, none of the reported predictive nomograms have included imaging features of the disease. Furthermore, the advantage of our nomogram is a higher overall accuracy (discriminant properties) and closer agreement between predicted and observed values (superior calibration). Estimating clinical utility of nomograms in prognosticating an outcome of intervention remains core value of translational research in precision medicine. Vickers and Elkin^22^ have introduced decision analysis curves estimating probabilities of benefits and harms that a diagnostic test or intervention can trigger at various thresholds. Addition of PIRADS score to nomogram achieved a higher net benefit of decisions making in comparison to leaving out PIRADS score as shown in the decision analysis curve constructed in the present study. The thresholds ranged from no upgrading of disease to all men having upgrading of disease following LRP.

### Clinical implications of the study findings

Predicting final histopathological Gleason score of prostate cancer remains a highly desirable information for physicians counselling men with localised prostate cancer for various modalities of treatment and long-term disease recurrence. At present, various nomograms are used mainly taking into consideration clinical factors such as age, pre-operative PSA level and number of biopsy cores involved with the cancer. Notwithstanding this, there is still a large histopathological discrepancy between biopsy and final radical prostatectomy Gleason score. The present study reports a nomogram based on pre-biopsy multiparameteric MRI grade (PIRADS score) of cancer alongwith other known clinical parameters. The nomogram clearly showed an improved prediction of final Gleason score and the findings have a large implications for clinicians and researchers in this area. We envisage that this and further research should take us close to precise prediction of final Gleason score of histopathology in prostate cancer and thereby an improved and informed decision making by stakeholders including patients in the management of localised prostate cancer. This will have huge benefits for improved GS prediction for men opting for active surveillance and focal therapy besides those opting for radical prostatectomy.

### Limitation of the study

There are limitations to our study. This is a single centre study with dedicated uro-radiologist and pathologist, and the rate of upgrading may be different in small centres. Moreover, overall accuracy of our model, although higher than previous was not perfect (90%). Additionally, performance of our model needs further validation in an external data set. Finally, the accuracy of our model could still be improved by integrating additional predictor variables, such as the novel genomic and other biomarkers^23,24^. The growing field of artificial intelligence and machine learning using radiomics approach may improve our ability to define tumour characteristics and classification. This, undoubtly may impact results of the study in the future. Finally, with emerging evidence supporting MRI facilitated biopsy targeting of suspicious areas using ultrasound, the rate of upgrading and the future implications for the practice may change^25^. There is an emerging evidence that targeted biopsy may improve our ability to narrow the upgrading gap between the biopsies and radical prostatectomy histology^26,27^; however no predictive nomogram information was available from both the studies. Our ongoing work through randomised intervention in MR/US fusion should be able to provide more information^28^

In Conclusions, PIRADS version 2 score of 4 or 5 are associated with an increased risk of biopsy Gleason Score upgrading. Pre-biopsy MRI and PIRADS score significantly and independently predict GS upgrading. If proven by external validation, this information should help in decision making by offering treatment options to men with localised prostate cancer.
